# The Role of Bidirectional VLC Systems in Low-Latency 6G Vehicular Networks and Comparison with IEEE802.11p and LTE/5G C-V2X

**DOI:** 10.3390/s22228618

**Published:** 2022-11-08

**Authors:** Stefano Caputo, Lorenzo Mucchi, Muhammad Ali Umair, Marco Meucci, Marco Seminara, Jacopo Catani

**Affiliations:** 1 Department of Information Engineering, University of Florence, 50139 Florence, Italy; 2 European Laboratory for NonLinear Spectroscopy (LENS), University of Florence, 50121 Sesto Fiorentino, Italy; 3 Istituto Nazionale di Ottica del CNR (CNR-INO), 50121 Sesto Fiorentino, Italy; 4 ARTES 4.0, Advanced Robotics and Enabling Digital Technologies & Systems, 56025 Pontedera, Italy

**Keywords:** visible light communication (VLC), 5G, 6G, intelligent transportation systems, bidirectional communication, vehicular communications

## Abstract

In this paper, we present very recent results regarding the latency characterization of a novel bidirectional visible light communication (VLC) system for vehicular applications, which could be relevant in intelligent transportation system (ITS) safety applications, such as the assisted and automated braking of cars and motorbikes in critical situations. The VLC system has been implemented using real motorbike head- and tail-lights with distances up to 27 m in a realistic outdoor scenario. We performed a detailed statistical analysis of the observed error distribution in the communication process, assessing the most probable statistical values of expected latency depending on the observed packet error rate (PER). A minimum attainable observed round-trip latency of 2.5 ms was measured. Using our dataset, we have also estimated the probability to receive correctly a message with a specific average latency for a target PER, and we compare it to the ultra-reliable low-latency (URLL) 5G communications service. In addition, a mobility model is implemented to compare the VLC and radio frequency (RF) technologies (IEEE802.11p, LTE, 5G) to support an automated braking systems for vehicles in urban platooning.

## 1. Introduction

In recent years, the integration of assisted driving and advanced communication technologies in modern vehicles has significantly increased, aiming at reducing fatalities and injury rates caused by accidents in urban scenarios [1]. Intelligent Transportation Systems (ITS) aim at increasing the safety and efficiency of the transportation system by interconnecting vehicles, humans and infrastructures through new communication technologies, so that vehicular units could readily share information on their dynamical state or information about surrounding environmental conditions [2,3,4]. However, according to a recent report by the World Health Organization (WHO) [5], more than 1 million people died annually and up to 50 million were injured in road accidents, despite the conspicuous efforts in introducing assisted reaction capabilities to critical events in new vehicles. Hence, the need for improvements in the modern transportation system safety clearly asks for further developments in advanced low-latency vehicular communication technologies, aimed at increasing the vehicle’s capability to promptly detect and respond to critical events through active and proactive safety protocols [6]. As a matter of fact, the fifth-generation (5G) communication standard has introduced, for the first time, a pillar called ultra reliable low latency (URLL), with the aim of providing mission-critical services, including road safety [7].

Before 5G-mobile communication systems, the Vehicle-to-Everything (V2X) communications were standardized by IEEE using wireless access for vehicular environments (WAVE) and the set of protocols of dedicated short range communication (DSRC). In particular, the radio frequency (RF) based physical layer in DSCR is defined in IEEE 802.11p, while the cellular-based competing technology for vehicular application (C-V2X) has been defined within Long-Term Evolution (LTE) technology as the LTE-V2X. Currently, the Third Generation Partnership Project (3GPP) is working on 5G-V2X standard, but in literature there are only some tests on the 5G network for vehicular applications [8].

Orthogonal frequency division multiplexing (OFDM) is often proposed to provide higher data rate in VLC systems. For example, in [9], a novel optical OFDM scheme for VLC systems is proposed to overcome the problem of power limitation and non-negative transmitted signal constraint of the lighting source. In [10], a hybrid OFDM-pulse time modulation (PTM) scheme where bipolar O-OFDM samples are converted into the digital PTM formats of pulse width modulation (PWM) and pulse position modulation (PPM) for the intensity modulation of light emitting diodes (LEDs) is proposed. In [11], a hybrid pre-coding technique-based peak-to-average power ratio (PAPR) reduction solution is proposed.

Vehicle to vehicle communication using VLC is a topic recently quite investigated by the scientific community. In [12], an analytical performance analysis of a car-to-car visible light communications system under different communication geometries during the daytime is reported. In [13], a driving test of a vehicle-to-vehicle (V2V) VLC prototype, with two cars driving on a highway, is performed. OFDM-based system is used to send data from one vehicle to the other (one way). In [14], a path loss expression as a function of distance and different weather conditions for a V2V link is considered. Ray tracing simulations are carried out to verify the accuracy. In [15], a market-weighted headlamp beam pattern model is employed to outline the relationship between the communication distance and bit error rate (BER) performance, considering both the line-of-sight (LOS) and non-line-of-sight (NLOS) links. In [16], a simple VLC system, based on commercial off-the-shelf LED headlamp and taillight, is presented and tested in several configurations in order to evaluate its compatibility with platooning. Both BER and latency are considered. In [17], a VLC-based vehicle localization method is proposed to overcome the problem of a critical brake in car platooning.

### 1.1. The Role of VLC in 6G

Latency performance of RF-based technology in vehicular applications is usually affected by the density of vehicles [18]. In this scenario, Visible Light Communication (VLC), which exploits LED-based lighting systems to encode information in the optical carrier through light intensity modulation schemes [19], recently raised as a prime candidate in the communication technology for the implementation of safety-critical protocols involving fast communication between vehicles [20] in Massive Machine-Type Communications (mMTC), as DSCR in urban scenario [21]. This interest was raised thanks to the large deployment of LED illumination systems in modern cars and motorbikes, and to the high directionality of the optical channel.

However, whilst most of the initial works in the field of VLC for ITS applications focused on the implementation of unidirectional links [22,23,24,25,26,27], also integrating VLC nd 5G technologies [28], only recently a preliminar work has been carried out on bidirectional VLC links [29]. The importance of a detailed analysis of latencies occurring in the message delivery process is highlighted in [30,31]. Bidirectional communication between vehicles is extremely important for mission-critical services such as assisted or automated driving. Ultra-low latency is a mandatory characteristic for future smart vehicles, and VLC is a promising technology for this scope. For example, if two vehicles continuously exchange data (e.g., the speeds), they can automatically keep a safe distance, allowing a secure braking in case a critical situation occurs.

In future 6G networks, the two technologies, RF and VLC, could coexist. An hybrid RF-VLC solution could be used to reduce the high density of RF users and to increase the range limitation of VLC technology [32]. While current 5G standard does not include VLC as an integrated technology for, e.g., vehicular communications, 6G is envisioned to include and integrate VLC as an enabling technology for killer applications, in particular in vehicular networks.

### 1.2. Our Contribution

This paper presents an experimental implementation of a bidirectional V2V communication based on VLC technology for the low latency exchange of messages. Experimental measurements have been carried out in a real urban scenario. Characterization of the transmission latency observed using a recently developed VLC system implementing a bidirectional link realized via modulation of light intensity emitted by motorbike head- and tail-lights [29] is reported. In such system, a bidirectional (round-trip) connection is established and performance is measured for relative distances between the two motorbikes up to 27 m and with data rates up to 57 kBaud. In addition, the performance of VLC as a technology for the assisted breaking system in modern cars has been evaluated and compared to RF-based technologies. A mobility model was used to evaluate the breaking distance of a modern car, based on the latency of the alert message arriving at the car.

The main contributions of this paper can be summarized as follows:

- First characterization of latency in a bidirectional VLC-based communication system for ITS application;
- Experimental measurements campaign have been carried out in a real urban scenario with real head- and tail- motorbike LED-based lamps;
- The results demonstrate that our low-cost system guarantees good latency performance, compared with the URLL 5G communication threshold [7];
- The simulation results using the experiments-based mobility model can highlight the benefit of VLC systems for future vehicular applications, as the communication interface currently planned by 6G.

The rest of the paper is organized as follows. Section 2 shows the measurements campaign setup, including the hardware equipment. Section 3 comments the experimental results and Section 4 shows the performance comparison between RF and VLC technologies and discusses the benefit of VLC in the urban platooning scenario. Finally, Section 5 concludes the paper.

## 2. Hardware Overview and Experimental Setup

The bidirectional VLC link is established performing an On-Off Keying (OOK) 0–200% modulation of the light intensity of a commercial headlamp (Qiilu QL04156, 12 V 160 mA current DC value) and of a tail light (Alchemy Parts, UPC: 5060502564597, 12 V 90 mA current nominal value) of a motorbike for distances up to 25 m with baud rates up to 57 kBaud [29]. Both lamps act as VLC transmission (TX) sources.

**Figure 1:**
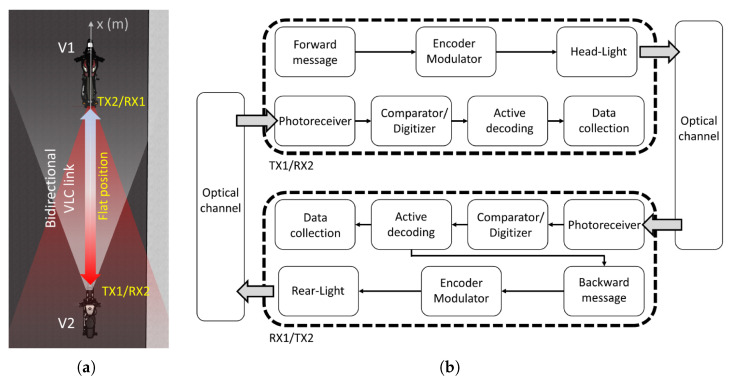
(**a**) Sketch of the bidirectional communication. The head-light (HL, TX1/RX2) and the rear light (RL, TX2/RX1) are placed facing each other at the same height. The TX1/RX2 sends a continuous data stream, the TX2/RX1 back replies only if the packet is correctly received. At the end, the TX1/RX2 fulfils a byte-wise comparison. (**b**) Block diagram of the experiment: the Forward message (in TX1/RX2 block) is fed into the head-light thanks a current modulator, which modulates the light with a Manchester encoding. The light travels through the optical channel and it is collected by a photoreceiver (in TX2/RX1 block). The collected analog signal is sent to a comparator/digitizer board, and the Arduino based microcontroller performs a byte-wise comparison between a pre-stored message and the resulting digital signal. If no wrong bytes are detected in a packet, the microcontroller counts it and generates a backward message, which is fed into the rear-light. Analogously to the forward message, the backward message travels back from the TX2/RX1 to the TX1/RX2, and it is compared with the stored reference message.

The experiment has been carried out in a realistic outdoor configuration in the central hour of the day under sunlight exposure with a luminous flux intensity of around 100 klux. Referring to Figure 1a, the lights are placed facing each other, at the same height of 75 cm from the ground, pointing forward (*Flat* configuration), which is the most realistic situation occurring in a real road scenario. A receiving system (RX) is placed besides each lamp, thus each lamp has one transmitter and one receiver. We named TX1 and RX2 the transmitter and receiver, respectively, of the first motorbike, while we named TX2 and RX1 the transmitter and receiver of the second motorbike, respectively. For a detailed analysis of the luminous pattern emitted in terms of the measured experimental SNR, considering the combination between the radiance map of the light sources and the FoV of the detectors, see [29]. Figure 1b shows the block diagram of our experiment. The first motorbike (TX1/RX2) is equipped with a low-cost, open-source microcontroller board (Arduino DUE), which sends a digital data stream of $10^{4}$ packets (9 byte each), through its head LED-based lights towards the second motorbike (TX2/RX1). The Manchester encoding is assured by a custom analog current modulator. The light collecting unit at the receiver is equipped with a customized version of a commercial variable gain photodetector (Thorlabs PDA100A2). The photodetector is equipped with an aspherical 2” uncoated lens (Thorlabs ACL50832U, focal length = 32 mm, resulting Field of View (FoV) = 17°) and a cylindrical shield (height = 75 mm, diameter = 85 mm, FoV = 60°), which is used to reduce the contribution of the stray light that affects the optical system [33]. The dimensions of the latter have been carefully selected in order not to limit the FoV of the optical system. The choice of the FoV of about 17° assures a sufficient FoV for the analysed scenario (Figure 1a) and at the same time reduces the contribution of any interference effect given by the contribution of other light sources or the sun that is out of sight. Furthermore, to reject the direct current (DC) sunlight component, a physical current DC-block stage is implanted into the photodetector before the first transimpedance stage. More detail about the receiver circuit is presented in [19]. The RX system is composed by the above mentioned light collecting unit together with a digitizing board and an Arduino based microcontroller, which performs the active decoding.

In case all packets of the message have been correctly received by the second motorbike (RX1), a message is generated and sent through the rear light back to the first motorcycle. Here, an analogous RX system, placed onboard to the headlights of the first motorbike, performs an active decoding of the received data stream, retrieving the number of correctly received packets (for further details see [19,29]). This allows us to measure both the Packet Error Rate (PER) and the latency for the bidirectional VLC link, which is a fundamental parameter for safety application [31]. We record the RX signal after a complete round-trip in the bidirectional VLC chain by using a 1 Gs/s digital oscilloscope (Siglent SDS1204X-E). The PER is measured as the ratio between the wrong packets received at the end of the round-trip and the number of sent packets ($10^{4}$), for distances up to 27 m.

**Figure 2:**
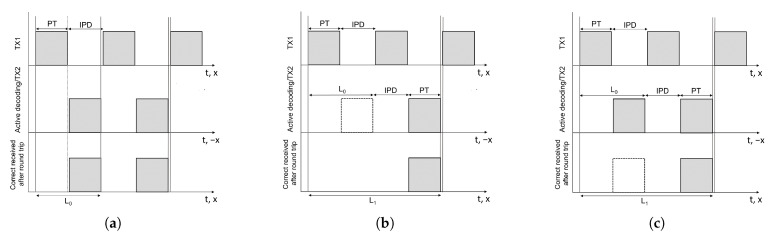
The three different time scales in each picture represent: the TX1, sending a continuous data stream of 10${}^{4}$ packets separated by an inter-packet delay (IPD) slightly greater then the packet time (PT) along the x direction; the TX2, after performing an active decoding and, if the packet is correct, back replies (−x direction) then, the final stage counts the correct received packets after a round trip. (**a**) If in all the round trip no packet is lost, the minimum latency is *L*${}_{0}$. The loss of the packet could happen both in the first path (**b**), and in the second path (**c**), in each case, the latency of the bidirectional link grows as $L_{N}=L_{0}+N_{Lost}\left(IPD+PT\right)$, where *N*${}_{Lost}$ is the number of consecutive lost packets in the final stage.

The latency could be defined as the time interval elapsed between the first bit of the transmitted packet and the last bit of the received packet after a round trip. As pointed out by Figure 2a, the TX1 transmits $10^{4}$ packets spaced by an *Inter-Packet Delay* (IPD) slightly greater then the *Packet Time* (PT) along the *x*-axis direction towards the second motorbike (TX2/RX1). If a perfect reception occurs, the TX2 replies back to the first motorbike and, again in the case of perfect reception, we find the minimum latency $L_{0}$. A Time Division Multiplexing (TDM) is used so that each return packet from TX2/RX1 is sent back to the first motorcycle (TX1/RX2) during the IPD occurring between two packets in the forward packet stream transmitted by TX1/RX2. In a bidirectional communication, the loss of a packet can occur both in the first transmission (Figure 2b) and in the second transmission (Figure 2c). In both cases, the latency grows as $L_{N}=L_{0}+N_{Lost}\left(IPD+PT\right)$, where $N_{Lost}$ is the number of consecutively lost packets in RX2 after a round trip. In safety-critical applications, the characterization of the clustering of lost packets is a fundamental aspect [30,31], as it determines the most probable latency value (*Statistically Averaged Latency*—SAL) corresponding to a certain PER in the communication. Indeed, assuming to have a data stream with a certain PT, IPD and PER = 0.5, several situations could arise depending on the size of error packets clusters and their occurrence. For example, the case where good packets and bad packets are evenly alternating is very different from the case where an initial perfect reception of packets is followed by a continuous stream of error packets, forming a very large error cluster. Even intuitively, these cases could tremendously differ in terms of expected latency.

## 3. Experimental Results

A thorough post-analysis of data is carried out to retrieve the errors’ distribution observed in the packet delivery process considering the bidirectional communication. In particular, we analyzed the occurrences and sizes of clusters of errors for different PER values in order to estimate the statistical value of the latency occurring in the complete round-trip communication. By observing the positions of the packets over time containing at least one error, we can measure the latencies to receive each packet correctly. The latencies obtained in such way can be used to derive the probability to reach a target average latency. In other words, we aimed to derive the probability that a specific latency occurs, i.e., that the message is correctly received by that time. The procedure is similar to the one reported in [30] for an I2V implementation.

**Figure 3:**
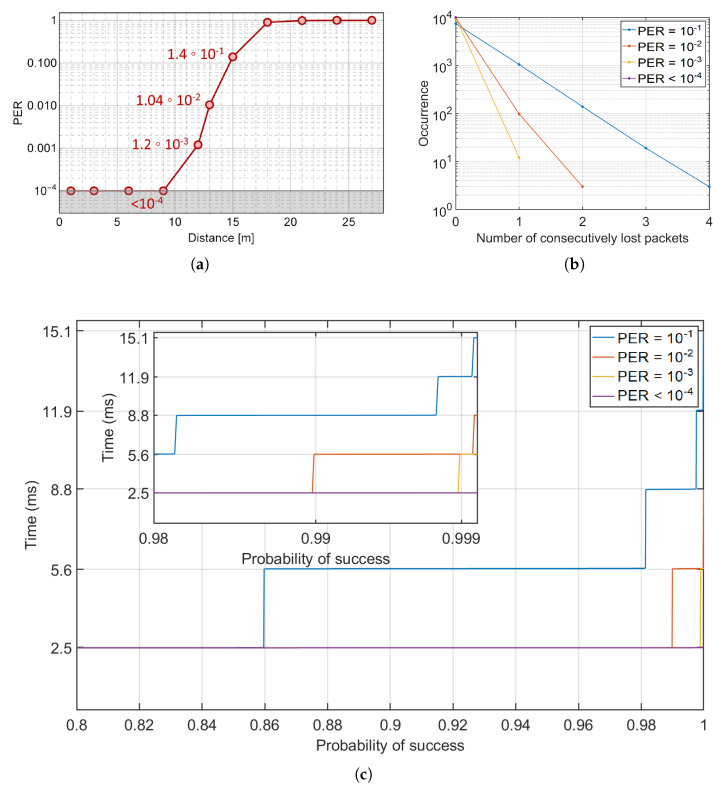
(**a**) PER of the VLC system with a baud rate of 57 kBaud. An error-free communication can be established for distances up to 9 m. To each symbol is associated an error bar, representing the standard deviation of the error. Horizontal and vertical error bars are smaller than the symbol mark. (**b**) Occurrences of the number of consecutively lost packets (errors cluster size) for different PER values. (**c**) SAL (ms) as a function of the success probability for different PER values.

The communication performance of our bidirectional VLC system is evaluated through PER measurements in a *Flat* configuration with a data rate of 57 kBaud. Since the number of transmitted packets is $10^{4}$, our PER limit corresponds to $10^{-4}$. Figure 3a shows the experimentally measured PER as a function of the distance between the motorbikes. The dots represent the statistical average over five repeated measurements, and both horizontal and vertical error bars, taken as standard deviations, are smaller than the symbols mark. The solid curve is a guide to the eye. As is well known, the PER depends monotonically from the Signal to Noise Ratio (SNR): as the distance increases the SNR decreases and, consequently, the PER increases, reaching a value of ≈0.1 at 15 m, while up to 9 m the PER is constantly below the $10^{-4}$ limit.

As discussed in Section 2, the statistical average latency (SAL) value is connected to the observed PER. The distribution of the number of consecutively lost packets during the experimental measurements is estimated and reported in Figure 3b for different PER values. The occurrences of the clusters of lost packets increases as the PER increases. The maximum observed cluster size is four (consecutive) packets with a PER of $1.4\times 10^{-1}$, that corresponds to a latency of 15.1 ms.

Following a procedure which has been recently employed in infrastructure-to-vehicle (I2V) applications [30], the number of consecutively lost packets has been sorted to obtain the SAL as a function of success probability, as depicted in Figure 3c. Table 1 shows the probability to reach a target SAL under a specific PER condition. As it can be observed, given a PER of $10^{-2}$, the SAL exceeds its minimum value (2.5 ms) with a probability of 1%, and with a probability of 0.1% for a PER of $10^{-3}$. Most significantly, our results demonstrate that even in the worst PER scenario (≃10${}^{-1}$), the probability of obtaining a successful bidirectional communication with a SAL lower than 2.5 ms is ≈86%, whilst in 98.2% of the cases the SAL is below 5.6 ms. Noticeably, even in this very unfavorable condition, the 5G threshold latency value (10 ms) for ITS services [7] is only exceeded with a probability lower than 0.3%. In all other PER cases, our VLC system shows a latency always below the 5G threshold with probabilities larger than 99.95%. Remarkably, in the case of PER = $10^{-4}$, a safety-critical message could be delivered in less than 2.5 ms with an error probability lower than $5\times 10^{-4}$.

It is important to remark that a significant aspect to be addressed in view of extensive deployment of bidirectional VLC links in ITS is represented by possible effects of unbalance in the quality between the forward and backward channels on the round-trip SAL values. We will provide further theoretical and experimental characterization of such aspects in future works.

## 4. Mobility Model, Performance Comparison and Discussion

In this section, a mobility model for the vehicles is provided and used to compare VLC and RF connection capabilities to enable fast braking automated systems. A platooning scenario is assumed to compare the braking capabilities of VLC- and RF-connected vehicles.

The mobility model is important to compare latency performance of RF communication standards and the proposed VLC system and to evaluate the limits of VLC in typical vehicular applications. In particular, we focused on the platooning case in an urban scenario, where the high density of vehicles makes RF communication not reliable enough to assure high-safety performances [18], mostly due to channel interference.

We considered two vehicles running over a road in a platooning situation. We aim to calculate the safety distance (the initial distance before the braking starts) which allows the vehicles to avoid collision. The latency of the communication link between vehicles is critical to start braking on-time and avoid collisions. Platooning in this case means that each vehicle is obligated to follow the previous vehicle, witth no possibility to change line. This is a frequent situation, in particular in urban areas. We aim to find which is the safety distance that two consecutive vehicles have to maintain in order to avoid collisions in case the first one suddenly starts a critical braking. When many vehicles are involved, it is important that each vehicle is connected to the previous and the following ones since this is what impacts the collision.

The implemented model simulates the dynamic trajectory of two vehicles during platooning. The model is used to define the minimum safety distance between the vehicles for different velocities of the vehicles. Different braking methods between the vehicles are compared: human reaction and automated/assisted braking systems based on communication technologies. The communication technologies that we considered are: RF standards such as 802.11p and C-V2X (LTE-based and 5G-based) and the proposed VLC system. The speed of the vehicles is assumed to be the same and both vehicles are assumed to be equipped with an automated braking system, activated by an alert message coming from the other vehicle.

To obtain the dynamic model of the two vehicles’ motion in the platooning scenario, we split the motion in two phases:

**Phase** **1.** The first vehicle starts to brake and its velocity decreases following the equation of uniformly decelerated motion. In the meanwhile, the second vehicle continues to move following the equation of linear motion with constant speed. This step lasts until the automated braking system engages.
**Phase** **2.** After the latency time of the communication system, the automated braking system engages and the second vehicle starts to brake following the equation of uniformly decelerated motion. The first vehicle continues to brake as in the previous point.

The deceleration value *a* used in uniformly decelerated motion is linearly proportional to gravitational acceleration *g*

(1) a=−μg

when μ is the friction coefficient. The friction coefficient is usually 0.7 for a road in good condition. The safety distance (sd) was evaluated as the difference between the braking distances of the two vehicles. To assure that a collision between the two vehicles does not occur, we set the safety distance sd as

(2) $$sd=\max\{sd_{phase_{1}},\;sd_{phase_{2}}\}$$

where $sd_{phase_{1}}$ and $sd_{phase_{2}}$ are the safe distances derived from Phase 1 and Phase 2 described above, respectively.

**Figure 4:**
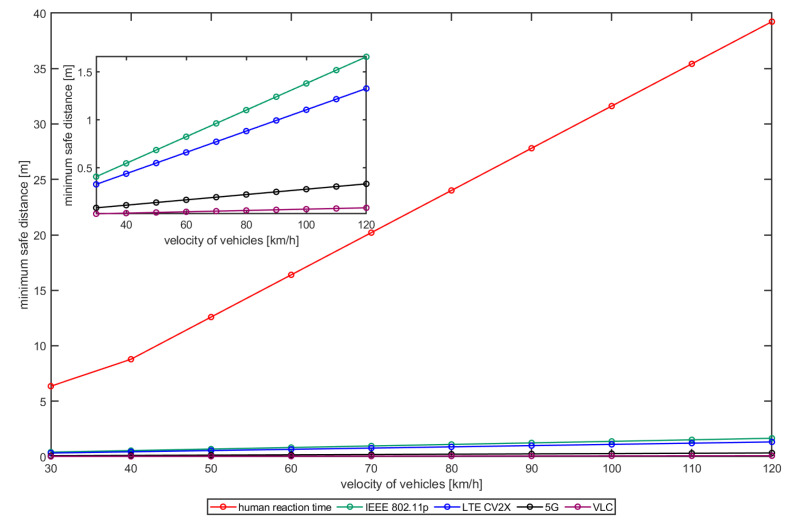
Minimum safe distance sd in platooning use case for different velocity of the two vehicles.

In Figure 4, the sd as a function of the vehicles velocity in a platooning scenario is demonstrated. The safety distance is mainly affected by the reaction time of the second vehicle. The human reaction time is equal to 1.37 s [34], while with the RF or VLC communication system the reaction time is the latency time to receive the alert message correctly from the other vehicle, which activates the automated braking system.

To define the latency of the RF technologies for vehicular communications, the experimental results in [8] was used. In particular, the latencies results:

- 50 ms for IEEE802.11p standard;
- 40 ms in C-V2X based on LTE network;
- 10 ms in C-V2X based on 5G network.

For the VLC communication system, we considered SAL =2.5 ms, as this value is granted with probability >99.95% for distances up to 9 m (see Figure 3a,c). As it can be observed in Figure 4, the safety distance between two vehicles (even with speed equal to 120 km/h) equipped with a VLC communication system could ideally be reduced to less than 0.1 m even in highway speed conditions.

The results of Figure 4 has been summarized in Table 2. The minimum safety distance significantly decreases with the VLC communication technology compared to others. Although safety distances are very small also for RF technologies, the VLC system provides one magnitude less.

**Figure 5:**
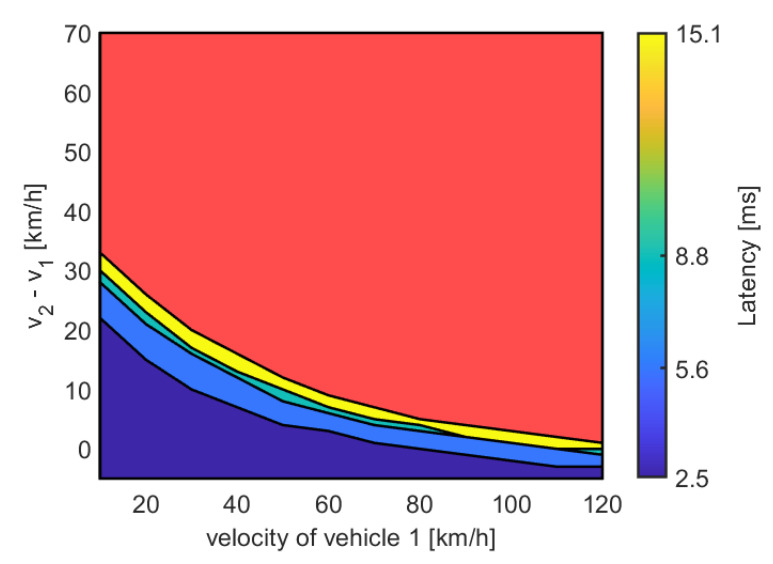
Latency of a VLC bidirectional connection as a function of the speed of the first vehicle ($v_{1}$ in the *x*-axis) and the speed difference between vehicles ($v_{2}-v_{1}$ in the *y*-axis). The red zone shows the speeds configuration so that a VLC connection cannot be established between the two vehicles. In the red zone, the latency is not defined since in that zone the two vehicles cannot support a stable connection.

The same mobility model is used to evaluate the performance of the proposed VLC system for two vehicles with different initial velocity. In this case, the two vehicles are assumed not to be connected yet. As it can be derived from Figure 3a, the VLC system experiences high error rate as the distance increases. Thus, if the safety distance of the vehicles is more than 15 m, vehicles cannot setup a connection, i.e., the safety distance has been calculated for every latency value (2.5, 5.6, 8.8, 11.9 and 15.1 ms) and if the safety distance is more than the distance in which the latency is ensured (see Figure 3a), the VLC communication between vehicles cannot be ensured with probability > 99.95%. The maximum difference between speeds that allows the connection between vehicles, and thus ensures braking, has been evaluated and the results are shown in Figure 5. The red zone in Figure 5 means that the value of the speed of the first vehicle and the value of the speed of the second vehicle do not guarantee the connection between the vehicles. For example, if the first vehicle has a speed of 60 km/h (*x*-axis in Figure 5), the speed of the second vehicle should not differ more than 10 km/h (*y*-axis in Figure 5) to setup a bidirectional connection by means of VLC signals. Figure 5 tells us also that if the first vehicle has high speed (120 km/h) the second one should have about the same speed to connect, and anyway with “high” latency (≈15 ms).

## 5. Conclusions

In this work, a first characterization of latency times in a bidirectional VLC link in a realistic outdoor scenario for V2V applications is presented. In particular, our system includes real motorbike LED head- and tail-lights as VLC sources, and experimental measurements have been conducted in an outdoor urban scenario. We characterized the communication performance of the system in terms of PER up to 27 m for a baud rate of 57 kBaud. Our results demonstrate an error-free bidirectional communication for distances up to 9 m, and a PER ≤0.1 for distances up to 15 m.

The analysis reported in this paper shows that our bidirectional V2V system is capable of establishing a VLC link with a minimum attainable bidirectional latency as low as 2.5 ms at 57 kBaud, yet granting latencies of the order of 5 ms for distances up to 10 m between vehicles. We highlight that this value is compatible with 5G URLL pillar for ITS, including road safety. We also present a mobility model aimed at comparing the braking distance attainable if two moving vehicles are equipped with VLC, 5G-URLL, LTE-V2X, and IEEEE802.11p communication technologies, respectively. Our model, in combination with our data, shows that VLC technology attains a reduction of the braking safety distance in a platooning scenario by nearly one order of magnitude as compared to what, e.g, IEEE802.11p RF-based systems, could allow. Our results pave the way towards the integration of VLC technology as a reliable communication platform in future ITS services, opening for future works aiming to provide more insight into further relevant aspects, such as tests in real driving conditions, effects of atmospheric conditions on performances of bidirectional VLC systems, or effects of relative unbalance between the forward and backward channel quality on the bidirectional round-trip latency.

## Figures and Tables

**Table 1:** Maximum expected SAL values as a function of PER, and their associated probabilities.

PER	Probability to Achieve a SAL Value				
	≤ 2.5 ms	≤ 5.6 ms	≤ 8.8 ms	≤ 11.9 ms	≤ 15.1 ms
$10^{-4}$	> 99.95%				
$10^{-3}$	99.9%	> 99.95%			
$10^{-2}$	99%	99.91%	>99.95%		
$10^{-1}$	86%	98.2%	99.7%	99.91%	> 99.95%

**Table 2:** Safety distance (m) for different vehicles speeds and communication technologies. Simulations are carried out with the mobility model (Section 4) in case of platooning.

Vehicles Speed	Safety Distance [m] for Platooning Scenario				
	VLC	5G	LTE	IEEE 802.11p	Human Reaction Only
40 km/h	0.03	0.11	0.44	0.55	8.78
60 km/h	0.04	0.17	0.66	0.82	16.40
80 km/h	0.06	0.22	0.88	1.10	24.01
100 km/h	0.07	0.28	1.10	1.38	31.62
120 km/h	0.08	0.33	1.33	1.66	39.23

## Data Availability

Not applicable.
